# Cutaneous sarcoidosis: clinico–epidemiological profile of 72 patients at a tertiary hospital in São Paulo, Brazil^☆^^☆☆^

**DOI:** 10.1016/j.abd.2019.06.004

**Published:** 2019-11-30

**Authors:** Mariana Fernandes Torquato, Marcella Karen Souza da Costa, Marcello Menta Simonsen Nico

**Affiliations:** Dermatology Department, Hospital das Clínicas, Faculdade de Medicina, Universidade de São Paulo, São Paulo, SP, Brazil

**Keywords:** Brazil, Epidemiology, Granulomatous disease, chronic, Sarcoidosis, Skin manifestations

## Abstract

**Background:**

Sarcoidosis is a multisystem disease of unknown cause that is characterized by the presence of granulomas in various organs. Cutaneous involvement is common and the reported incidence has varied from 9% to 37%. Studies on cutaneous sarcoidosis in Brazil are lacking.

**Objectives:**

To describe the clinical and epidemiological aspects of patients with cutaneous sarcoidosis diagnosed at the Department of Dermatology of the University of São Paulo, from May 1994 to March 2018.

**Methods:**

Clinical data of patients with confirmed cutaneous sarcoidosis were retrospectively reviewed and classified according to gender, ethnicity, age at diagnosis, cutaneous presentation, systemic involvement and treatment.

**Results:**

Cutaneous sarcoidosis was diagnosed in 72 patients with a female predominance (74%). The mean age at diagnosis was 49.6 years and most of the patients were white (61%). Papules and plaques were the most common lesions. Systemic sarcoidosis was detected in 81% of patients, affecting mainly the lungs and thoracic lymph nodes (97%). Typically, cutaneous lesions were the first manifestation (74%). Systemic therapy was necessary for 72% of patients; the dermatologist managed many of these cases. Oral glucocorticoids were the most commonly used systemic medication (92%). The mean number of systemic drugs used was 1.98 per patient.

**Limitations:**

Insufficient data in medical records.

**Conclusions:**

This series highlights the dermatologist role in recognizing and diagnosing cutaneous sarcoidosis, evaluating patients for systemic disease involvement and treating the skin manifestations. Cutaneous sarcoidosis was once considered exceedingly infrequent in Brazil in comparison to infectious granulomatous diseases; however, the present series seems to suggest that the disease is not so rare in this region.

## Introduction

Sarcoidosis is a multisystem disease of unknown cause that is characterized by the presence of granulomas in various organs. Studies show that genetic susceptibility and environmental factors contribute to the development of an exaggerated immune response.^1^ The clinical course varies and, in half of the cases, the disease resolves spontaneously within two to five years. After five years, remission is much less likely.^2^ Lungs and thoracic lymph nodes are the most commonly affected organs in more than 90% of cases, but nearly every other organ can be affected.^3^

Cutaneous involvement is common and the reported incidence has varied from 9% to 37%.^2^, ^4^, ^5^, ^6^, ^7^ Skin manifestations can be classified into two categories: specific lesions have histopathological evidence of typical sarcoid granulomas; nonspecific lesions develop as a result of an inflammatory reaction pattern.^2^, ^4^, ^5^, ^6^, ^7^, ^8^, ^9^ Erythema nodosum is the most common nonspecific skin lesion, often found with acute sarcoidosis, and usually portends a good prognosis.^10^ Specific lesions are chronic, asymptomatic, and require therapy.^2^, ^4^, ^5^, ^6^, ^7^, ^8^, ^9^

Typical specific lesions include macules, papules and plaques, lupus pernio, and subcutaneous nodules (known as Darier-Roussy nodules). Less common manifestations include scar- and tattoo-associated lesions, and hypopigmented, angiolupoid, psoriasiform, erythrodermic, ulcerative, ichthyosiform, verrucous, or lichenoid forms.^2^, ^4^, ^5^, ^6^, ^7^, ^8^, ^9^

When it is clinically suspected, a skin biopsy is important to establish histopathologic evidence of sarcoidosis and to rule out other causes of granulomatous disease, by demonstrating the presence of microorganisms in tissue cultures.^11^ Histopathology reveals non-necrotizing granulomas composed of mononuclear histiocytes, which can form into giant cells and have minimal surrounding lymphocytic inflammation.^4^ Once cutaneous sarcoidosis is confirmed, evaluation for systemic involvement is mandatory.^2^, ^11^

Therapy is usually directed at the organ most severely affected, which often helps cutaneous lesions. For patients with isolated cutaneous sarcoidosis or patients with the severe cutaneous disease, a stepwise approach is recommended with local therapies, immunomodulators, and systemic immunosuppressants.^12^

Sarcoidosis is considered infrequent in Brazil; its aspects have not been analyzed since 1976, when a review of 40 cases was conducted.^13^ The objective of this study was to characterize the clinical and epidemiological features of cutaneous sarcoidosis at a tertiary hospital in São Paulo, Brazil.

## Methods

After receiving approval from the institutional review board, a retrospective study was conducted analyzing the clinical records of 72 patients diagnosed at Department of Dermatology at the University of São Paulo, Brazil, from May 1994 to March 2018.

Diagnosis of cutaneous sarcoidosis was based on compatible clinical picture, histopathologic evidence of sarcoid granulomas on skin biopsy, and exclusion of other granulomatous diseases by special stains for microorganisms and cultures. All patients underwent a systemic evaluation including history with a complete review of organ systems, physical examination, chest radiography (radiographical staging for pulmonary sarcoidosis [RSPS]),^2^, ^11^ pulmonary function tests, ophthalmologic evaluation, standard hematological and biochemistry profiles, electrocardiogram, and tuberculin skin test. Further investigation was performed as necessary, according to the individual clinical picture. Patients were excluded if their medical charts were not available.

Clinical data were retrospectively reviewed to obtain the following: gender; age at diagnosis; ethnicity; morphology and distribution of cutaneous lesions; extracutaneous involvement; initial manifestation; and treatment used. After that, descriptive statistics were used to summarize the information.

## Results

Of the 74 patients initially collected with a confirmed diagnosis of cutaneous sarcoidosis, two patients for whom there was no clinical information were excluded. The clinical characteristics of the remaining 72 patients are summarized in table 1. The male to female ratio was approximately 1:2.8, with 19 males (26%) and 53 females (74%). The mean age at diagnosis was 49.6 years (range 28–69 years). The majority of patients were white (61%).

**Table 1:** Clinical characteristics of 72 patients with cutaneous sarcoidosis.

Characteristics	Patient number	Percentage (%)
*Sex*		
Male	19	26
Female	53	74

*Ethnicity*		
White	44	61
Afro-descendant	28	39

*Age, years*		
Mean	49.6	
Range	28–69	

*Specific lesions*		
Plaques	32	44
Papules	30	42
Subcutaneous	11	15
Lupus pernio	8	11
Angiolupoid	4	6
Hypopigmented	2	3
Tattoo lesions	2	3
Scar lesions	1	1
Psoriasiform	1	1
Lichenoid	1	1
With ≥2 types	20	28

*Special sites*		
Nail	3	4
Oral cavity	2	3
Scarring alopecia	2	3
Genital	1	1

*Distribution*		
Head and neck	50	69
Face	44	61
Face only	16	22
Nose	21	29
Upper extremities	38	53
Lower extremities	26	36
Trunk	25	35
Lesions at ≥2 locations	40	56

**Figure 1:**
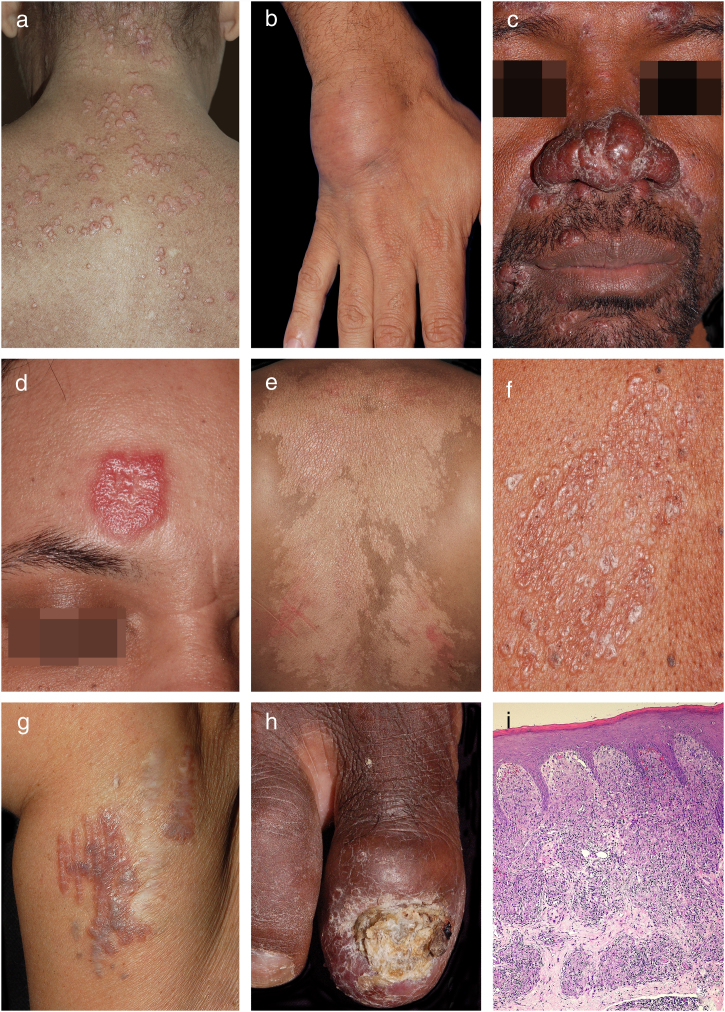
Clinical examples of cutaneous sarcoidosis: A, Papular form; B, Subcutaneous form; C, Lupus pernio; D, Angiolupoid; E, Hypochromic form; F, Lichenoid lesions; G, Ancient scar lesions; H, Periungual infiltration with osteitis and nail dystrophy; I, Granulomas composed predominantly of epithelioid histiocytes (Hematoxylin & eosin, x40 ).

Specific cutaneous lesions observed included plaques in 32 patients (44%), followed by papules in 30 (42%), subcutaneous nodules in 11 (15%), lupus pernio in eight (11%), angiolupoid sarcoidosis in four (6%), hypopigmented lesions in two (3%), psoriasiform lesions in one (1%), and lichenoid lesions in one (1%). Scar- and tattoo-associated sarcoidosis lesions were seen in one (1%) and two (3%) patients, respectively (Fig. 1). Twenty patients (28%) had two types of lesions. Eight patients had lesions at special locations, such as the nails (3), oral cavity (2), scalp (specific cicatricial alopecia) (2), and genitals (1). The head and neck were the most commonly affected sites, with lesions in 50 patients (69%). Forty-four patients (61%) presented facial involvement. Lesions affected only the face in 16 patients (22%) and 21 (29%) had lesions on the nose. Lesions were present in the upper extremities in 38 patients (53%), the lower extremities in 26 (36%), and the trunk in 25 (35%). Fifty-six patients (40%) demonstrated lesions in two or more locations.

Systemic sarcoidosis was detected in 58 patients (81%), and cutaneous lesions were the first presentation in 43 (74%) of these (Table 2). Diagnosis of extracutaneous sarcoidosis occurred before cutaneous disease in 15 patients (26%). Pulmonary sarcoidosis was the most frequent systemic manifestation, affecting 56 patients (97% of patients with systemic compromise). According to the RSPS,^1^ 25 patients had stage I pulmonary sarcoidosis (lymphadenopathy alone), 21 had stage II (pulmonary infiltration with lymphadenopathy), two had stage III (pulmonary infiltration without lymphadenopathy), and eight had Stage IV (pulmonary fibrosis). Other commonly involved organs were the kidneys in eight patients (14%), the extrathoracic lymph nodes in eight (14%), and the eyes in seven (12%). Of the patients with kidney disease, five had only asymptomatic hypercalciuria and three had nephrolithiasis. The most common ocular manifestations were uveitis and lacrimal gland enlargement in five and two patients, respectively. Two patients (3%) had cardiac sarcoidosis. The systemic disease had affected the liver, spleen, pancreas, gastrointestinal tract, upper respiratory tract, muscles, bones, and articulations in one patient each, respectively. Sarcoidosis remained confined to the skin in 14 patients (19%).

**Table 2:** Characteristics of extracutaneous involvement.

Characteristics	Patient number	Percentage (%)
Extracutaneous involvement	58	81
Chest	56	97
Stage 1	25	43
Stage 2	21	36
Stage 3	2	3
Stage 4	8	14
Kidney	8	14
Extrathoracic lymphadenopathy	8	14
Ocular	7	12
Heart	2	3
Hepatic	1	2
Spleen	1	2
Pancreas	1	2
Gastrointestinal tract	1	2
Muscles	1	2
Upper respiratory tract	1	2
Articular	1	2
Bones	1	2
First manifestation		
Cutaneous	43	74
Extracutaneous	15	26

Fifty-two patients (72%) required systemic therapy and 12 patients (17%) had only local therapy (Table 3). Three patients were lost to clinical follow-up after diagnosis, one had spontaneous remission, and four had not yet started treatment. Of the patients who were treated only with local therapy, topical glucocorticoid was used in ten, intralesional glucocorticoid in five, and topical calcineurin inhibitor in three. In half of these cases, association with local therapies was necessary. Oral glucocorticoid represented the most frequently used systemic medication in 48 patients (92%), followed by antimalarial drugs in 28 (54%), methotrexate in 18 (35%), azathioprine in four (8%), leflunomide in two (4%), tetracycline-class antibiotics in two (4%), thalidomide in one (2%), and infliximab in one (2%). Seventeen patients (33%) received oral corticosteroids as monotherapy. The mean number of systemic drugs used in each patient during the follow-up period was 1.98. Systemic therapy was directed to cutaneous lesions and, therefore, managed exclusively by the dermatologist in 24 patients (46%).

**Table 3:** Treatment of patients with cutaneous sarcoidosis.

Characteristics	Patient number	Percentage (%)
Only local therapy	12	17
Topical glucocorticoid	10	83
Intralesional glucocorticoid	5	42
Topical calcineurin inhibitor	3	25
Systemic therapy	52	72
Oral glucocorticoid	48	92
Antimalarial	28	54
Methotrexate	18	35
Azathioprine	4	8
Leflunomide	2	4
Tetracycline-class antibiotics	2	4
Thalidomide	1	2
Infliximab	1	2

## Discussion

Although the prevalence of sarcoidosis in Brazil has yet to be established, it is estimated to be lower than 10 cases/100,000 population.^14^ Older Brazilian dermatologists claimed that cutaneous sarcoidosis was exceedingly rare in Brazil; it was usually stated that “a case of cutaneous sarcoidosis is simply a misdiagnosed case of leprosy or tuberculosis.” It appears that its incidence might be slowing increasing over the last few decades.^2^ Incidence by sex and the median age at diagnosis in the present series coincide with statistics from other countries. However, most of the present patients were white (61%), contrary to the classic higher prevalence in Afro-Americans.^2^, ^4^, ^15^

Cutaneous lesions led to the detection of extracutaneous sarcoidosis in 74% of patients with systemic involvement, a figure supported by other studies.^16^, ^17^

According to several series, the most common specific lesions of sarcoidosis are papules.^18^, ^19^, ^20^ However, plaque-type sarcoidosis was the most frequent presentation in the current study (44%), followed by papules (42%). This proportion was also found in recent series from Lebanon and Taiwan.^21^, ^22^ Plaques may arise *de novo* or from a confluence of papules. When compared to papules, plaques tend to have a deeper infiltration and are more likely to resolve with permanent scarring. The presence of plaques has been associated with a chronic disease course.^18^, ^19^, ^20^

Specific subcutaneous nodules (not erythema nodosum) were seen in 15% of the present patients, a higher frequency than in other studies.^8^, ^18^, ^20^

Lupus pernio, the most characteristic lesion of cutaneous sarcoidosis, usually follows a chronic course and often coexists with sarcoidosis of the upper respiratory tract.^7^ In the current series, eight patients presented lupus pernio, all with associated systemic disease, often severe and with the involvement of many organs. Only one patient had upper respiratory tract involvement. Scar- and tattoo-associated sarcoidosis lesions were diagnosed in one and two patients, respectively. These lesions can be misdiagnosed as hypertrophic scars or keloids.^7^ Less frequent specific lesions such as angiolupoid, hypopigmented, psoriasiform, and lichenoid sarcoidosis were detected in a few patients.

Facial involvement was present in 61% of patients; a similarly high proportion has been reported by other studies.^22^, ^23^ In 40% of cases, lesions affected two or more locations.

The authors’ routine initial procedure for the diagnosis of sarcoidosis included skin histopathology (with negative stains for microorganisms), chest radiography, and a negative tuberculin skin test. This protocol was sufficient to diagnose systemic sarcoidosis in most patients. After diagnosis, the basic assessment included ophthalmological evaluation as well as hematological and biochemical profiles (urine and serum calcium levels, liver and renal function tests), electrocardiogram, and pulmonary function tests. Additional tests were requested as needed. Serum angiotensin-converting enzyme levels are not measured at this study's facilities. Only 60% of patients with sarcoidosis have increased levels of this enzyme, and it is not specific to the disease.^7^

If systemic sarcoidosis cannot be demonstrated in a patient with skin granulomas, a long-term follow-up should be undertaken. In the present series, systemic sarcoidosis was detected in 81% of patients; in almost all, it could be demonstrated immediately after and as a consequence of the diagnosis of cutaneous disease. Pulmonary sarcoidosis was the most common systemic manifestation, affecting 97% of the cases. Lymphadenopathy was the most frequent radiological finding (82%), followed by pulmonary infiltration (41%) and fibrosis (14%). Other commonly involved organs were the kidneys (14%), the extrathoracic lymph nodes (14%), and the eyes (12%). Except for renal involvement, which was present in a greater proportion in the current study, other organ involvements were found at similar rates to those previously described.^1^ A higher frequency of renal manifestations might be due to a possible underdiagnosis of asymptomatic hypercalciuria in previously studied patients and emphasizes the need to measure not only the serum calcium level but also the urinary calcium.^11^, ^12^

Fourteen patients presented only cutaneous manifestations; it is controversial in the literature whether these patients may ultimately develop additional organ system involvement or if their disease will be restricted to the skin.^12^

Topical and intralesional steroids were the used local therapies in the patients. These drugs are considered as a first-line therapy for cutaneous sarcoidosis with limited or mild disease, or as an adjunct to help control cutaneous disorders when the extracutaneous disease is under adequate control. Topical calcineurin inhibitors, which have a low overall side-effect profile and are indicated for limited skin disease, can also be used.^12^, ^24^ Of the 12 patients (17%) who were treated only with local therapy, five presented isolated skin lesions and seven had mild pulmonary disease, not requiring systemic management.

For patients with severe disease or those who do not respond to topical therapy, oral corticosteroids are first-line systemic therapy. In the event of a relapse or if the signs persist, steroid-sparing agents should be instituted alone or with a low-dose corticosteroid.^12^, ^25^ In the present series, oral glucocorticoids were the most frequently used medication (92%), at times as monotherapy (33%). Antimalarials (54%) and methotrexate (35%) were also used. Other steroid-sparing medications included azathioprine (4 patients), leflunomide (3), tetracycline-class antibiotics (3), and thalidomide (1). Infliximab has been recently used for recalcitrant sarcoidosis.^25^ This drug was prescribed for one patient who presented lupus pernio and had severe systemic and cutaneous disease after therapeutic failure with other combined regimens.

The mean number of systemic drugs used in each patient during the follow-up period was 1.98, which confirms that most patients needed to receive one to two different medications to achieve disease control. Systemic therapy was directed toward cutaneous lesions and, therefore, managed exclusively by the dermatologist in 24 patients (46%). These patients presented only cutaneous compromise (seven) or had mild pulmonary involvement (17).

## Conclusion

The clinical and epidemiological findings in the present study were mostly similar to those published in the literature. Its limitations include retrospective design and, in some cases, lack of detailed data in the patient records. Compared with data from the literature, an increased incidence of renal impairment (hypercalciuria) was observed. Despite the small sample size, it represents the largest Brazilian series on this subject, suggesting that sarcoidosis may not be so infrequent in Brazil. Although there are many endemic granulomatous infectious diseases in this country, sarcoidosis certainly stands out as an important diagnosis to be considered.

## Financial support

None declared.

## Authors’ contribution

Mariana Fernandes Torquato: Statistical analysis; conception and planning of the study; composition of the manuscript; collection, analysis, and interpretation of data; critical review of the literature.

Marcella Karen Souza da Costa: Statistical analysis; conception and planning of the study; composition of the manuscript; collection, analysis, and interpretation of data; critical review of the literature.

Marcello Menta Simonsen Nico: Approval of the final version of the manuscript; participation in the design of the study; intellectual participation in the propaedeutic and/or therapeutic conduct of the studied cases; critical review of the literature.

## Conflicts of interest

None declared.
